# Supplementary material for: Coupled Genome-Wide DNA Methylation and Transcription Analysis Identified Rich Biomarkers and Drug Targets in Triple-Negative Breast Cancer
Source: Cancers (Basel). 2019 Nov 4;11(11):1724. doi: 10.3390/cancers11111724 (PMC6896154; doi:10.3390/cancers11111724)
Supplement: Supplementary file 1 [file cancers-11-01724-s001.zip › cancers-634306-suppl-final/Supplementary methods and materials.docx]

Supplementary Materials: Coupled Genome-Wide DNA Methylation and Transcription Analysis Identified Rich Biomarkers and Drug Targets in Triple-Negative Breast Cancer

Maoni Guo, Siddharth Sinha and San Ming Wang

Supplementary Methods

Protein structure and FDA approved drugs preparation

The protein structures 5IKV for gene PTGS2 (COX-2) was downloaded from protein data bank (www.rcsb.org) and optimized using the UCSF chimera [1]. The FDA approved drugs identified for the *COX-2* were screened for its binding with the respective protein structure (5IKV). The binding pattern determination for the drugs with the protein structure have been performed using the molecular docking and Molecular Dynamics Simulations (MDS).

Molecular Docking

Binding pattern determination for the *COX-2*-inhibitor complex was performed using Autodock 4.0 [2]. Autodock combines energy evaluation through precalculated grids of affinity potential employing various search algorithms to find the suitable binding position for a ligand on a given protein [2]. Binding site for drug was chosen around Arg-513 side chain which includes all amino acid residues in the active site [3]. Grid size was set to 60 x 60 x 60 grid points (x, y and z), with spacing between grid points kept at 0.375 Å. The Lamarckian genetic algorithm was chosen to search for the best conformers. Standard docking protocol was applied which is based on population size of 150 randomly placed individuals, a maximum number of 250,000 energy evaluations, a mutation rate of 0.02, a crossover rate of 0.80 and an elitism value of 1. Twenty-five independent docking runs were carried out for each inhibitor and cluster tolerance was kept at 1.0 Å.

The binding free energy evaluation by Autodock 4.0 includes intermolecular energy (van der Waals energy, hydrogen bonding energy, desolvation energy, and electrostatic energy), internal energy and torsional energy. The first two items build up docking energy; the first and third items compose the binding energy. The difference in binding free energy of three different classes of drugs provides further insights into their binding mode with *COX-2*.

Molecular Dynamics Simulations

Based on docking results, MDS of active site *COX-2*-inhibitor complex was carried out using Gromacs 5.1 suite of programs using gromos force field [4]. Each of the complexes was placed in center of 90*90*90 Å cubic box and solvated by SPC/E water molecules [4]. The gromacs topology file for ligands was generated using the PRODRG2 server. The time constant for berendsen temperature coupling and berendsen pressure coupling were both set at 0.1. The environment was set to 300K and 1 bar. All of the complexes were energy minimized using steepest descent method. Further, a 30 ps position restraining simulation was carried out to restrict the movement of the protein in the simulation. For the long-range electrostatic interactions, Particle Mesh Ewald (PME) electrostatic was used. The cut-off for coulomb interaction and Vander Waal interaction were set to 1.0 nm and 1.4 nm respectively. The LINCS algorithm was used for all bond constraints.

We examined the mobility of drugs in active site through 10 ns MD simulation in order to understand how the binding affects the *COX-2* structure. The selection was based on the optimal binding conformation obtained by drug in the group. The optimal conformation was ascertained on following criteria: docking energy, binding conformation, and hydrogen bonding with active site residues. The drug enzyme complex was analyzed through following parameters: Root mean square deviation (RMSD), Root mean square fluctuations (RMSF), Radius of gyration (Rg), Number of hydrogen bond (NH-bond).

RMSD: RMSD is commonly used as an indicator of convergence of the structure towards an equilibrium state. It measures low values of deviation for native and variant average structures over a period of time.

RMSF: RMSF measures flexibility of polypeptide chain by calculating the fluctuation of C-alpha atoms coordinating from their average position thereby determining residue flexibility of protein segments between native and bounded protein complex.

Rg: Radius of gyration (Rg) shows the distance of the atoms of the structure from either its center of gravity or an axis. It is a measure of compactness for each protein structure i.e. native and bounded with drug.

NH-bond: The presence of a hydrogen bond is inferred from the distance between a donor-H-acceptor pair and the donor-H-acceptor angle. As hydrogen bonds are important in maintaining steady configuration of protein, NH bond analysis of native and bounded form of the protein helps to determine the liaison between flexibility and NH bond formation


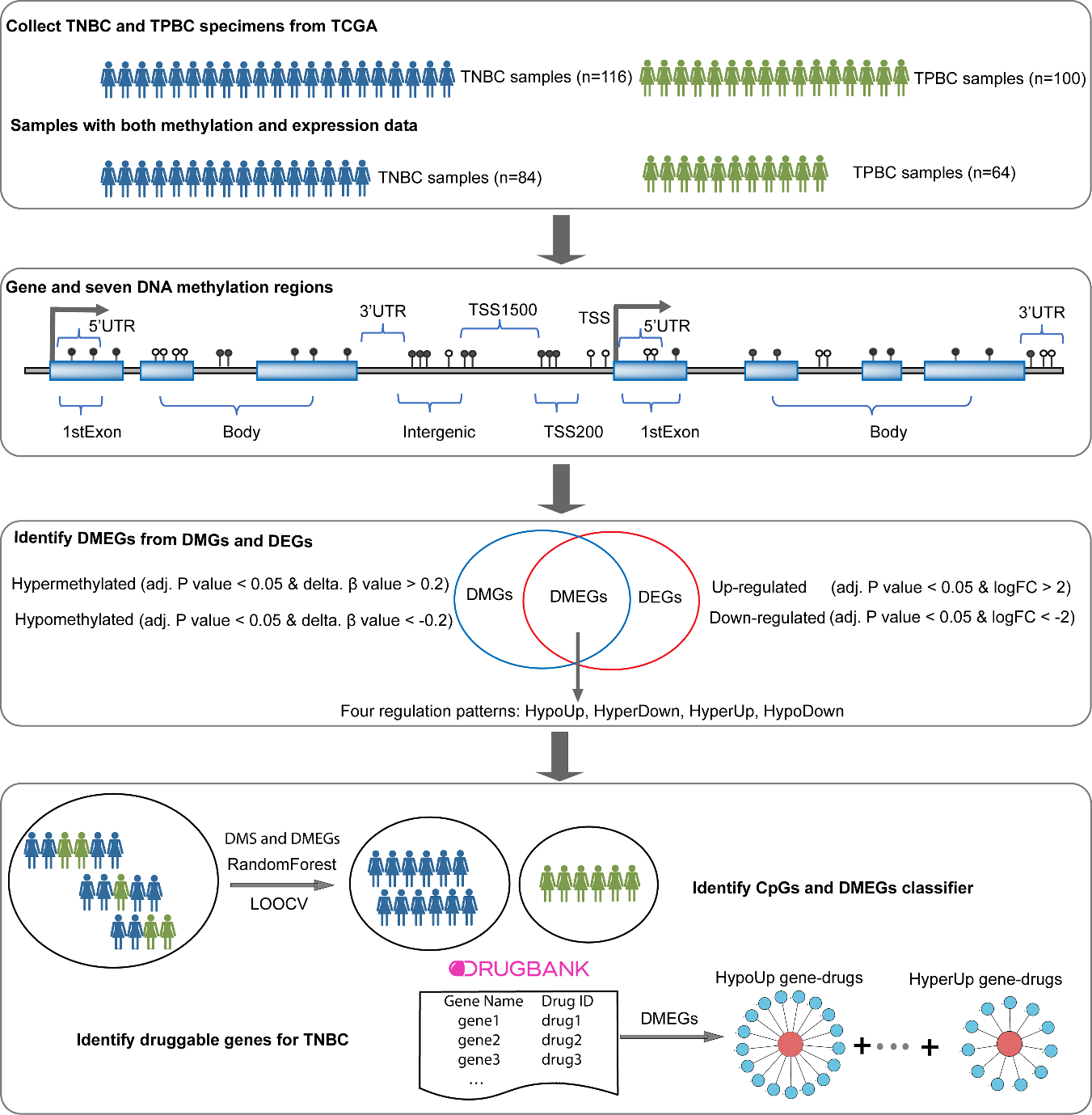


**Figure S1.** Flowchart of the analytical process. DMEGs were identified by comparing DMGs and DEGs between TNBC and TPBC. Candidate therapeutic targets were identified from DrugBank database. See detailed description in Materials and Methods.


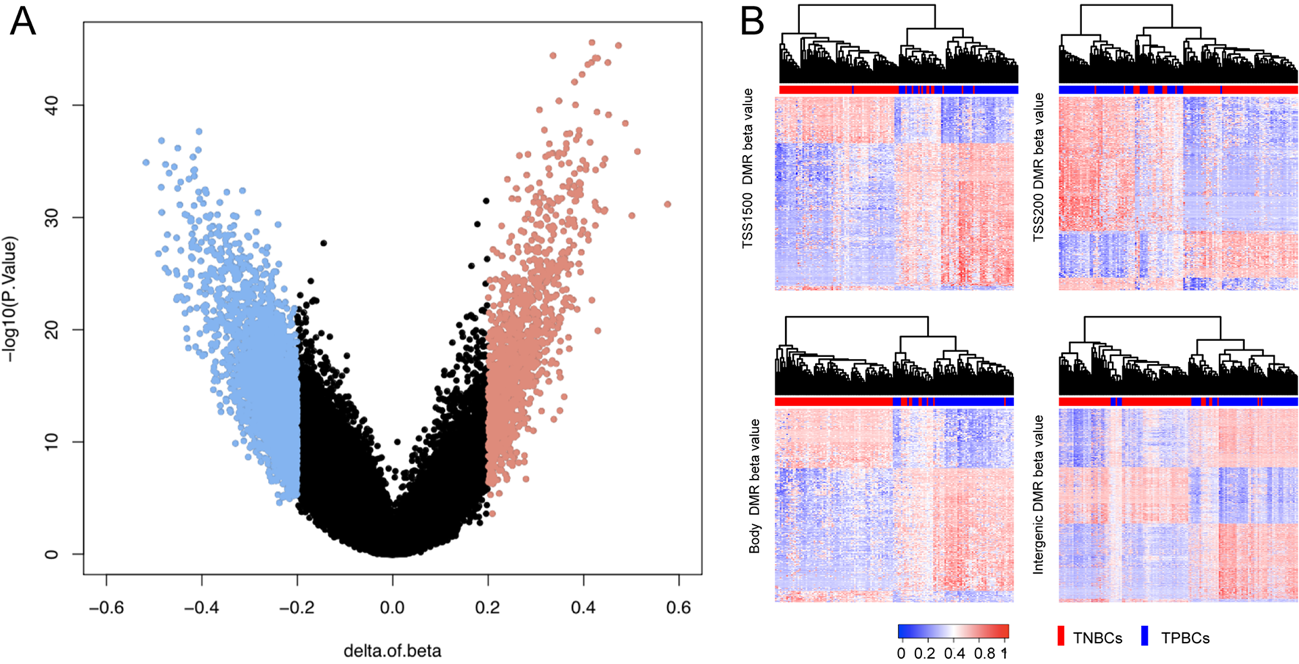


**Figure S2.** DMGs in TNBC. (**A**) The volcano plot of DMGs in intergenic region. The red and blue dots represent the significantly hyper- and hypomethylated DMGs with adj. *p* value < 0.05 and delta. β value > 0.2 or delta. β value < −0.2, respectively. (**B**) Heatmaps showing the methylation profile of 84 TNBCs and 64 TPBCs across DMGs in TSS1500 (*n* = 396), TSS200 (*n* = 394), gene body (*n* = 270) and intergenic (*n* = 2,063) regions.


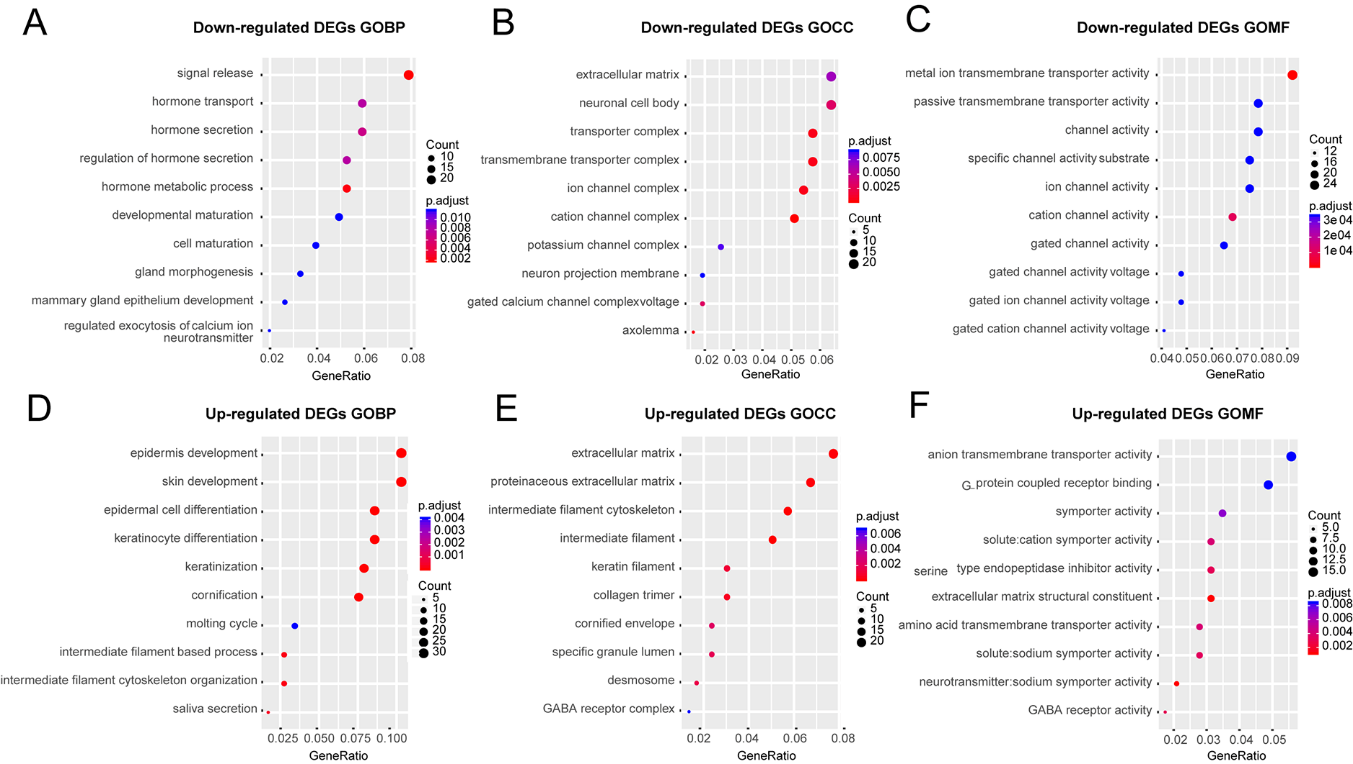


**Figure S3.** DEGs in TNBC. (**A**–**C**) The top 10 significant GO functional enriched BP, CC and MF terms for 378 down-regulated DEGs. (**D**–**F**) The top 10 significant KEGG functional enriched pathways for 332 up-regulated DEGs.


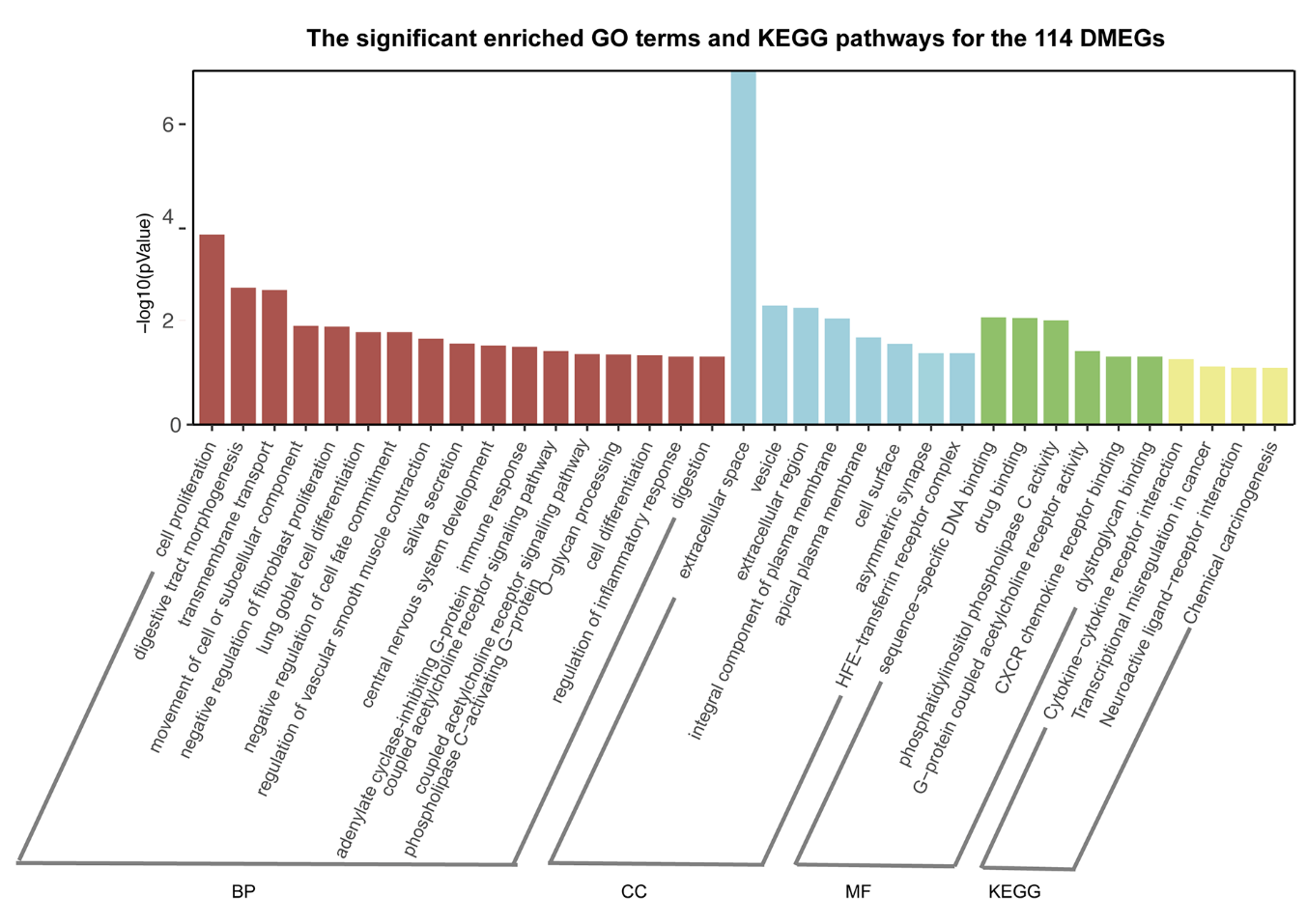


**Figure S4.** The significant enriched GO terms and KEGG pathways for the 114 DMEGs.


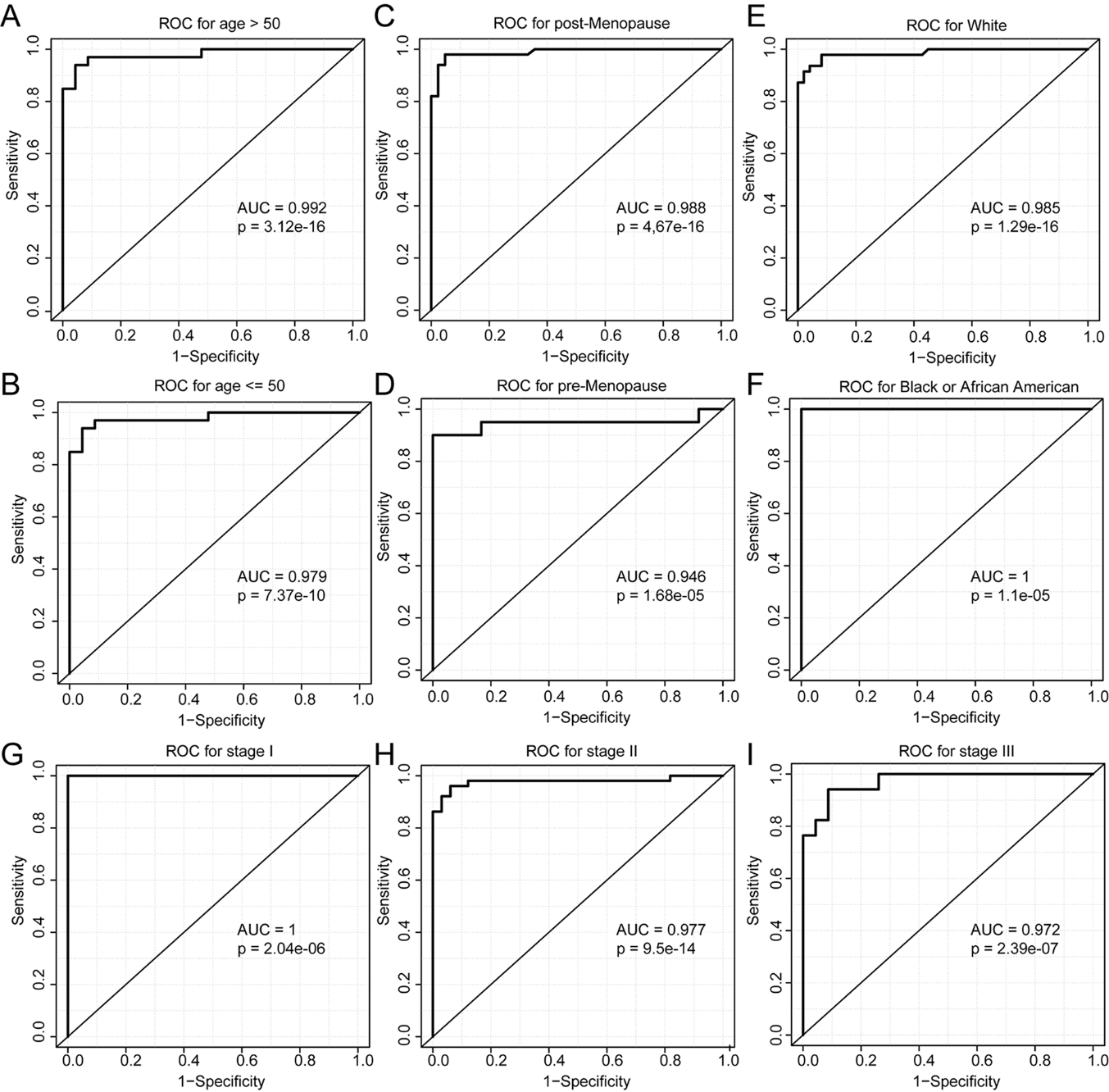


**Figure S5.** Stratification analyses of the 114-DMEG predictor with available age, stage, menopause and ethnicity information for all TNBC patients. (**A**) ROC curve for TNBC patients with age > 50, (**B**) ROC curve for TNBC patients with age ≤ 50. (**C**) ROC curve for post-Menopause TNBC patients (**D**) ROC curve for pre-Menopause TNBC patients. (**E**) ROC curve for White TNBC patients, (**F**) ROC curve for Black TNBC patients. (**G**) ROC curve for TNBC patients with stage I, (**H**) ROC curve for TNBC patients with stage II, (**I**) ROC curve for TNBC patients with stage III and IV.


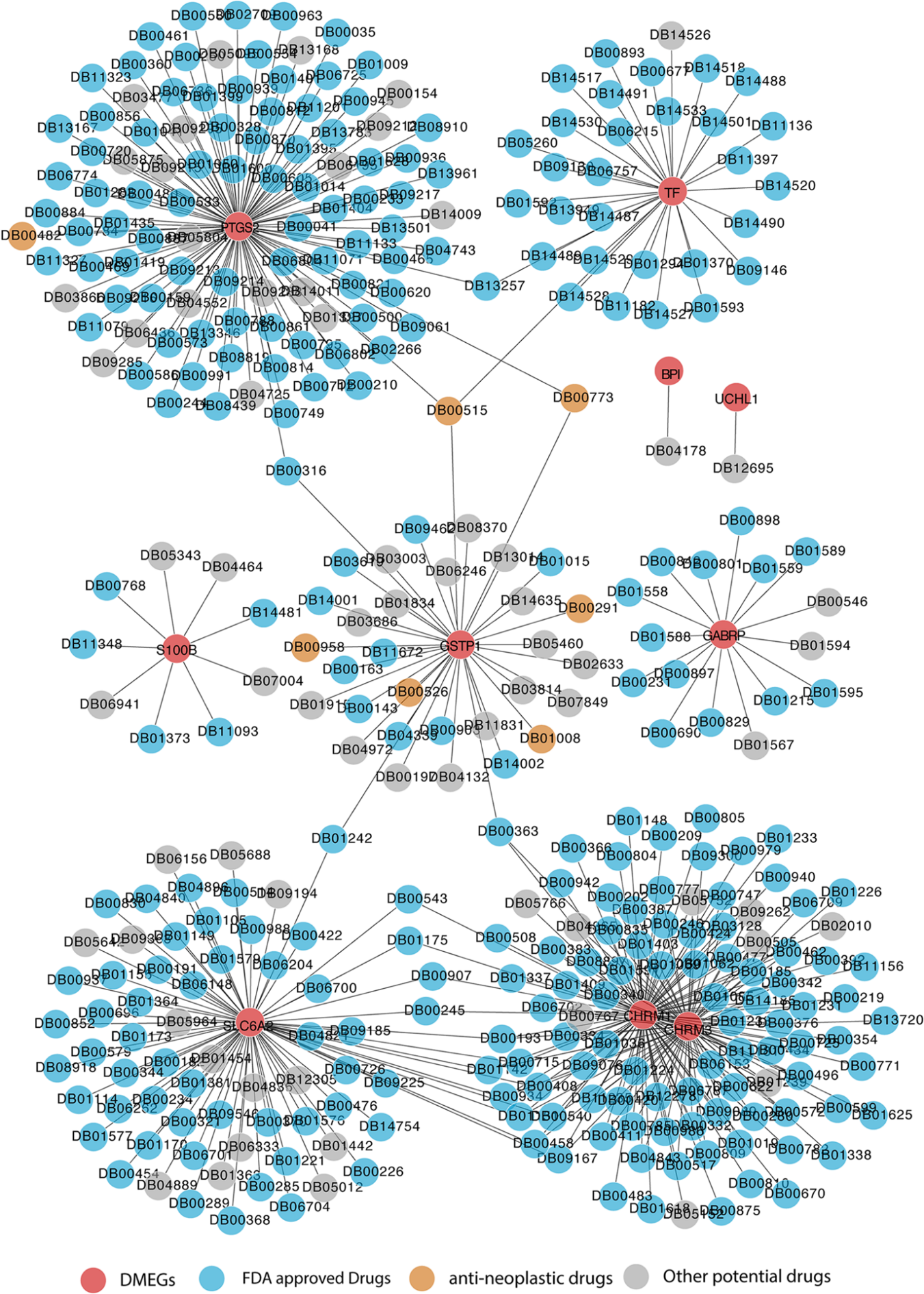


**Figure S6.** Drug and DMEG interactions predicted with our pipeline.

Please View the Supplementary Tables at the Excel Files.

**Table S1.** The characteristics of TNBC and TPBC samples. **Table S2.** DMGs harboring DMRs in TSS1500, TSS200, Body and Intergenic regions between TNBC and TPBC. **Table S3.** DEGs in TNBC. **Table S4.** DMEGs in TNBC. **Table S5.** Drugs targeting 10 DMEGs predicted with our pipeline.

**References**

1. Pettersen, E.F.; Goddard, T.D.; Huang, C.C.; Couch, G.S.; Greenblatt, D.M.; Meng, E.C.; Ferrin, T.E. UCSF Chimera--a visualization system for exploratory research and analysis. *J. Comput. Chem.* **2004**, *25*, 1605–1612, doi:10.1002/jcc.20084.

2. Morris, G.M.; Huey, R.; Lindstrom, W.; Sanner, M.F.; Belew, R.K.; Goodsell, D.S.; Olson, A.J. AutoDock4 and AutoDockTools4: Automated docking with selective receptor flexibility. *J. Comput. Chem.* **2009**, *30*, 2785–2791, doi:10.1002/jcc.21256.

3. Padhye, S.; Ahmad, A.; Oswal, N.; Dandawate, P.; Rub, R.A.; Deshpande, J.; Swamy, K.V.; Sarkar, F.H. Fluorinated 2'-hydroxychalcones as garcinol analogs with enhanced antioxidant and anticancer activities. *Bioorg. Med. Chem. Lett.* **2010**, *20*, 5818–5821, doi:10.1016/j.bmcl.2010.07.128.

4. Hess, B.; Kutzner, C.; van der Spoel, D.; Lindahl, E. GROMACS 4: Algorithms for Highly Efficient, Load-Balanced, and Scalable Molecular Simulation. *J. Chem. Theory Comput.* **2008**, *4*, 435–447, doi:10.1021/ct700301q.

| 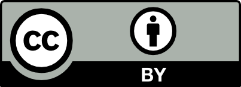 | © 2019 by the authors. Licensee MDPI, Basel, Switzerland. This article is an open access article distributed under the terms and conditions of the Creative Commons Attribution (CC BY) license (http://creativecommons.org/licenses/by/4.0/). |
| --- | --- |
